# Transgenic models of cardiac arrhythmias and sudden death

**DOI:** 10.3389/fphys.2013.00060

**Published:** 2013-03-28

**Authors:** Carol Ann Remme

**Affiliations:** Department of Clinical and Experimental Cardiology, Academic Medical Center, University of AmsterdamAmsterdam, Netherlands

Cardiac arrhythmias are associated with a wide range of clinical problems, ranging from relatively benign (a)symptomatic heart rhythm alterations to life-threatening ventricular arrhythmias associated with sudden cardiac death. In most cases, atrial and ventricular arrhythmias are acquired, i.e., secondary to cardiac (structural) pathologies, including valvular disease, heart failure, cardiomyopathy, coronary artery disease, and myocardial infarction. Mechanisms underlying arrhythmogenesis in these conditions have been extensively investigated in the last decades (Janse, 2004; Rubart and Zipes, 2005). In a subset of patients, however, cardiac arrhythmias occur secondary to congenital or inherited alterations in electrophysiological properties of cardiomyocytes. Cardiac arrhythmias secondary to hereditary genetic disorders are increasingly recognized as a cause for sudden cardiac death in the general population (Perrin and Gollob, 2013). Mutations in a large number of genes encoding either ion channels, proteins involved in cell–cell coupling, or other proteins directly or indirectly influencing cardiac electrophysiology have been identified in affected patients (Amin et al., 2010; George, 2013). Widespread genetic testing has in some cases allowed for development of genotype-specific treatment strategies (Hofman et al., 2010; Ackerman et al., 2011). However, in-depth studies of the mechanisms underlying the observed electrophysiological abnormalities are often limited in patients, and *in vitro* studies in heterologous expression systems may not adequately reflect the endogenous cardiomyocyte environment. As reviewed in this Research Topic of *Frontiers in Cardiac Electrophysiology*, transgenic mouse models incorporating the genetic defect in question may be successful in studying genotype-phenotype correlations and may provide important insight into the underlying electrophysiological, biophysical, and molecular mechanisms.

The availability of genetically engineered transgenic mice has allowed for detailed investigation of the *in vivo*, *ex vivo*, and *in vitro* electrophysiological consequences of human mutations associated with arrhythmias. However, as reviewed by Kaese and Verheule, certain cardiac electrophysiological properties are inherently different between mouse and man (Kaese and Verheule, 2012). The authors conclude that transgenic mouse models may nevertheless successfully mimic human arrhythmia syndromes although observations regarding arrhythmia mechanisms may only be extrapolated to the human situation with caution. Grubb and co-workers provide an overview of studies on mice deficient for the potassium channel interacting protein KChIP2, a component of the transient outward potassium current (*I*_to_) (Grubb et al., 2012). These studies have provided increased insight into the multiple actions of this promiscuous accessory protein, and KChIP2 is now known to modulate numerous potassium channels in addition to L-type calcium channels and possibly sodium channels (Grubb et al., 2012). The cardiac conduction system is another area where genetic defects in ion channels have been associated with arrhythmias and sudden cardiac death. Hyperpolarization-activated Cyclic Nucleotide-gated (HCN) channels, which are responsible for the pacemaker funny current *I*_f_, are located specifically within the conduction system. A range of transgenic mouse models of different HCN isoforms has shed light on the role of *I*_f_ on pacemaking and atrio-ventricular conduction, as reviewed by Bucchi et al. (2012) and Aránega et al. (2012). In other models, genetic defects in certain ion channels widely expressed throughout the myocardium were found to have specific electrophysiological consequences within the specialized conduction system, which may promote arrhythmias (Aránega et al., 2012). In particular, mice with genetic alterations in the *Scn5a* gene encoding the cardiac sodium channel have provided insight into the role of this channel in both sinus node dysfunction and progressive cardiac conduction disease, as detailed by Huang et al. (2012). The various available *Scn5a* transgenic mouse models (in addition to mice lacking specific sodium channel auxillary β-subunits) have furthermore been instrumental in clarifying the biophysical alterations underlying Long QT syndrome type 3, Brugada syndrome, and cardiac conduction disease, inherited conditions associated with sodium channel mutations (Derangeon et al., 2012). Transgenic mice also allow for in-depth investigation of the interaction between (abnormalities in) cardiogenesis and arrhythmias. This is exemplified by Franco and colleagues who review the role for the transcription factor PITX2 in cardiac (in particular pulmonary vein) development and its link with atrial arrhythmias including atrial fibrillation (Franco et al., 2012). The many existing mouse models of atrial fibrillation and their usefulness for studying the pathways and mechanisms underlying this complex arrhythmia is further discussed by Riley et al. (2012). Defects in cytoskeletal signaling pathways have also been associated with atrial fibrillation, as shown by studies in mice with a heterozygous deficiency for the adapter protein Ankyrin-B (Smith et al., 2012). *Ankyrin-B*^+/−^ mice have further provided fundamental insight into the role of ankyrins in sinus node disease and ventricular arrhythmias (Smith et al., 2012). Finally, the use of murine transgenic and targeted models of desmosomal proteins associated with arrhythmogenic right ventricular cardiomyopathy (ARVC) has been instrumental in investigating disease etiology and progression in addition to the complex interaction between cell adhesion defects and arrhythmogenesis in this inherited syndrome (Lodder and Rizzo, 2012).

Apart from transgenic mouse models, novel techniques are increasingly recognized as useful in studying electrical roles of novel identified genes, and electrophysiological effects of specific mutations in the native cardiomyocyte environment. Zebrafish (*Danio rerio*) are relatively easy to study and genetically modify, but crucial differences exist between zebrafish and human cardiac electrophysiology (Verkerk and Remme, 2012). Nevertheless, ion channel disorders related to repolarization disorders have been successfully modeled in zebrafish; their applicability in studying depolarization disorders and calcium-related arrhythmias is however as yet unclear (Verkerk and Remme, 2012). More recently, it has been shown that human-induced pluripotent stem cell-derived (iPSC) cardiomyocytes may recapitulate disease phenotype in Mendelian cardiac rhythm disorders (Hoekstra et al., 2012). Although the interpretation of electrophysiological data from iPSC-derived cardiomyocytes should be done with caution (due to their immature phenotype), they are considered a promising tool for studying pathophysiology, genotype-phenotype relationship, and pharmacology in cardiac arrhythmia syndromes (Hoekstra et al., 2012).

In summary, the collection of 12 articles presented in this Research Topic provides an overview of the knowledge and insight obtained from various transgenic and targeted models of cardiac electrophysiology, and how further research in this field may be of additional benefit for the future identification, risk-stratification, and treatment of patients with inherited cardiac arrhythmias and sudden death.
